# Enhancing tobacco quality and flavor through cold plasma treatment: structural, chemical, and sensory modifications

**DOI:** 10.3389/fmolb.2025.1724715

**Published:** 2025-11-26

**Authors:** Xiujuan Xu, Min Wang, Qingzhao Shi, Shan Liu, Weiping Yang, Chunqiang Yang, Ji Ma, Renqi Wang

**Affiliations:** 1 Zhengzhou Tobacco Research Institute of National Tobacco Corporation, Zhengzhou, China; 2 School of Food and Biological Engineering, Shaanxi University of Science and Technology, Xi’an, China

**Keywords:** cold plasma, nontargeted analysis, LC-MS, lipids, flavor, non-thermal treatment

## Abstract

Cold plasma (CP) treatment has emerged as a promising green processing technology for enhancing the quality of tobacco by modifying its physical structure and chemical composition. This study investigated the effects of CP on Zimbabwe and Yunnan tobacco, focusing on its impact on sensory attributes, microstructure, and molecular composition. CP treatment, optimized at 100 kV for 1 min, enhances both the aroma and smoke smoothness, increasing sensory scores from 6 to 7. Scanning electron microscopy (SEM) revealed that CP treatment induced surface disruption, creating irregular pores and increasing the pore density, which could enhance drying kinetics. Nontargeted metabolomics analysis reveals 43 putatively annotated metabolites which are significantly varied after CP treatments (i.e., fold change >3 and q-value <0.05), which correlated with improved sensory characteristics. Among these annotated compounds, substantial changes in the lipidomic profile is identified, with specific reductions in highly unsaturated and ether-linked phospholipids. These findings suggest that CP treatment selectively modifies surface lipids, potentially improving tobacco flavor by reducing harshness and enhancing aroma release. Furthermore, the study confirmed the role of CP in reducing excess surface lipids, as demonstrated using soy lecithin-doped tobacco as a model system. Overall, CP offers significant potential for enhancing tobacco flavor and quality, with implications for more sustainable and efficient post-harvest processing.

## 1 Introduction

Cold plasma (CP) is a non-thermal, environmentally friendly, and residue-free green processing technology which inactivates microorganisms and suppresses enzyme activity through a combination of physicochemical mechanisms, including high-energy electron bombardment, ozone oxidation, and ultraviolet photolysis (Xu et al., 2024). The ionized plasma contains a variety of high-energy reactive species, including radicals, charged particles, excited-state species, and electromagnetic radiation quanta (Sruthi et al., 2022). These effects help minimize the degradation of food sensory quality and nutritional value. On a physical level, CP treatment was shown to disrupt the structural integrity of the samples, creating numerous irregular pores. It also weakened hydrogen bonding between water molecules and the food matrix, thus accelerating the drying process and promoting the outward diffusion of internal molecules (Bao et al., 2021; Chen X. et al., 2024). From a chemical perspective, CP treatment reduced the 2,2-diphenyl-1-picrylhydrazyl (DPPH) radical scavenging activity of samples. It also induced a variety of chemical reactions, such as C-O covalent bond cleavage (Cao et al., 2020), deesterification (Momeni et al., 2018), and the fragmentation and rearrangement of fatty acid chains, which facilitate the release and perception of aroma compounds. Biologically, CP treatments could significantly reduce microbial populations. In an observation of fresh tilapia fillets stored for 12 days after CP treatment, total viable bacteria, *Pseudomonas* spp., and Enterobacteriaceae levels were found significantly lower than those in the untreated control group (Wang et al., 2022). In peanuts, CP treatments reduced Aspergillus flavus spore counts and aflatoxin B1 (AFB1) content by 56.22%–75.27% and 86.34%–99.17%, respectively, after storage of 6 days, demonstrating strong antimicrobial efficacy (Zhao et al., 2025). Meanwhile, CP treatments have marginal impacts on the flavor of foods. For example, CP-treated coconut water retained a volatile profile highly similar to that of fresh coconut water after 7 days of storage, especially preserving key aroma notes such as buttery and popcorn-like characteristics (Xu et al., 2024). In summary, CP exerts synergistic effects across physical, chemical, and biological domains, significantly enhancing food stability, extending shelf life, and improving sensory quality to a certain extent. These multifaceted benefits highlight its strong potential for broad application in food processing.

CP can be generated at atmospheric pressure, such as dielectric barrier discharge (DBD), corona discharge, gliding arc discharge, and radio frequency discharge (Yang X. et al., 2024). Among these methods, DBD is especially suitable for food industry due to its simple operation, large discharge area, uniform discharge characteristics, flexible process parameters, and good performance at room temperature (Anuntagool et al., 2023; Murtaza et al., 2024). Adjusting key parameters like voltage and treatment time allows DBD to enhance antimicrobial efficacy and drying efficiency while avoiding thermal degradation of sensitive compounds. This helps preserve or even improve the sensory and biochemical properties of raw materials. For example, Pan et al. reported that increasing DBD voltage enhanced the generation of reactive oxygen and nitrogen species, leading to fungal spore deformation and DNA damage, and significantly improving microbial inhibition on apricot surfaces (Pan et al., 2023). Ni et al. observed that higher DBD voltage strengthened electroporation in lotus pollen, forming more and larger pores, accelerating the migration of internal moisture and substances. Meanwhile, ozone generated during discharge promoted the oxidative dehydrogenation of ferulic acid to vanillic acid, with conversion efficiency increasing with voltage (Ni et al., 2022). Dousti et al. found that the degradation of AFB1 in oat seeds was positively correlated with treatment time, as longer exposure generated more reactive oxygen species (Dousti et al., 2024). Seelarat et al. further demonstrated that extending the DBD pretreatment time significantly improved the drying performance of jackfruit slices. Moreover, an appropriate extension of treatment time effectively increased the contents of ascorbic acid, total phenolics, flavonoids, and polysaccharides, as well as the antioxidant capacity (Seelarat et al., 2024). Therefore, atmospheric DBD cold plasma exhibits strong potential for applications in microbial inactivation, efficient drying, and quality enhancement.

CP has recently been explored for its potential in improving the processing and storage quality of tobacco. As a natural flavoring plant with high economic value, tobacco is rich in diverse aroma compounds (Xu et al., 2025), complex microbial communities (Hu et al., 2025), and various active enzymes (Zhang et al., 2023). These constituents collectively contribute to its unique flavor profile. However, they also render tobacco highly susceptible to quality deterioration during storage and processing, including microbial spoilage, enzymatic browning, and component degradation coupled with storage risks due to excessive moisture (Zhao et al., 2022; Fu et al., 2024). Therefore, effective microbial inactivation and gentle moisture removal are essential for preserving tobacco quality, extending shelf life, and enhancing sensory attributes. Conventional tobacco treatments primarily include air-curing, sun-drying, and flue-curing. These aim to remove most moisture, improve appearance, and enhance aroma (Zou et al., 2019; Chen et al., 2021). While industrially mature and stable, these methods inevitably present significant drawbacks. High-temperature flue-curing often causes the volatilization and thermal degradation of heat-sensitive aroma compounds, leading to weakened characteristic aromas or reduced flavor complexity (Wang et al., 2025b). Concurrently, conventional drying has limited efficacy in inactivating endogenous microorganisms and fungal toxins, resulting in incomplete sterilization (Yang Y. et al., 2024). Additionally, their long drying cycles and high energy consumption constrain sustainable development. CP pretreatment has shown promise in addressing these limitations. Studies report that CP can reduce the natural drying time of tobacco midribs and whole leaves by 37.7% and 19.5%, respectively. It also lowers nicotine content, increases the carbohydrate-to-protein ratio, and alters the composition of the final product, suggesting substantial industrial potential (Khudyakov et al., 2022). Nonetheless, the underlying molecular mechanisms by which CP enhances tobacco flavor remain unclear and warrant further investigation.

In this study, CP was applied to Zimbabwe and Yunnan tobacco to investigate its potential for enhancing flavor quality. The effects of CP treatment were systematically examined at both physical and chemical levels. Scanning electron microscopy (SEM) analysis was employed to assess structural and surface area changes in tobacco leaves following treatment. Additionally, a liquid chromatography–high-resolution mass spectrometry (LC-HRMS)-based nontargeted metabolomics approach was used to explore the molecular alterations associated with flavor enhancement under varying CP voltages and treatment durations. This integrated analysis aims to provide a comprehensive understanding of how CP modulates the physical structure and chemical profile of tobacco, offering insights into its application for improving sensory attributes and product quality.

## 2 Materials and methods

### 2.1 Sample description and treatments

This study involved flue-cured tobacco leaves sourced from two representative geographical origins: Kunming (China) and Zimbabwe. All tobacco materials were sourced from the Zhengzhou Tobacco Research Institute of China National Tobacco Corporation, following the standardized criteria outlined in YC/T 210.6–2006, “Tobacco Leaf Code Part 6: Tobacco Leaf Grade”. Before CP treatment, moisture contents of the tobacco leaves were adjusted to approximately 25%. Then, the samples were equilibrated in a chamber maintained at a consistent temperature (i.e., 22 °C ± 2 °C) and humidity (i.e., 60% ± 5%) for 1 week. Soybean phosphatidylcholine and sphingomyelin, with purities of 98% and 99% respectively, were purchased from Acmec (China) and Macklin (China). The experimental design consisted of two treatment groups. The first group received exclusively DBD (SML-80kV; Suman Plasma Technology Co., Ltd., Nanjing, China) treatment. The operational parameters were set as follows: current intensity 50 mA, frequency 50 Hz, electrode gap distance 60 mm, with ambient air as the working gas. The highest working voltage of the instrument was 120 kV. To investigate the effects of CP treatment on tobacco leaves, we selected the maximum voltage (120 kV) and 80% of the maximum (100 kV). While previous studies typically set the CP treatment duration using DAD to 1 min, we also explored the effects of extended treatment durations of 4–6 min (Sun et al., 2023; Chen X. et al., 2024). Kunming tobacco samples were treated at 100 kV for 1 and 6 min, and at 120 kV for 1, 4, and 6 min. Zimbabwean tobacco samples were treated for 1 min at both voltage levels. Untreated samples from both origins served as controls. Soy phospholipids, a plant-derived lipid material, were selected to verify the ability of cold plasma (CP) to modify lipid composition. Commercial soy phospholipids (soy lecithin mixture; Sigma-Aldrich, ≥99% purity, containing mainly phosphatidylcholine, phosphatidylethanolamine, and phosphatidylinositol) were used as a model lipid material to simulate the surface lipid layer of tobacco leaves. Specifically, 4 mg of soy phospholipids were dissolved in 8 mL of ethanol and uniformly sprayed onto 30 g of Kunming tobacco shreds using an automatic sprayer commonly used for flavor addition in tobacco (N800-II, BAIZE INST Co. Ltd., Zhengzhou, China). The amount of soy phospholipids added was empirically determined based on the typical addition level of commercial flavor used in cigarettes (i.e., approximately 100 ppm). The lipid-supplemented tobacco was sealed in bags and allowed to equilibrate for 2 h at room temperature. Thereafter, dielectric barrier discharge (DBD) treatment was performed at 100 kV for 1 min. Subsequently, 1 g of the treated tobacco was ground into fine powder, mixed with 5 mL of 75 vol% ethanol, and subjected to ultrasonic extraction for 40 min. The extract was then filtered to obtain the tincture. For nontargeted analysis, 1 mL of the tincture was mixed with 4 mL of methanol, centrifuged at 10,823 × *g* for 10 min at 4 °C, and the resulting supernatant was transferred to HPLC vials for subsequent analysis.

### 2.2 Sensory evaluation

Sensory evaluation of the tobacco shred samples was conducted in accordance with the Chinese tobacco industry standard YC/T 415-2011, “Tobacco In-Process—Sensory Evaluation Methods.” The processed tobacco shreds were manufactured into standard cigarettes for sensory assessment under blind conditions. The evaluation panel consisted of ten qualified experts, each with more than 5 years of experience in the tobacco industry. Samples were evaluated using the overall circular smoking method. Twelve sensory attributes were assessed, including aroma quality, aroma volume, volatility, off-flavor, concentration, strength, smoke smoothness, glomeration, irritation, dryness, cleanliness, and aftertaste sweetness. Each attribute was rated on a 9-point scale, and the final score was calculated as the average of all individual attribute scores. An increase of 0.5 points in a specific attribute was regarded as an improvement, while an increase of 1 point indicated a discernible improvement.

### 2.3 SEM characterization

A small amount of tobacco shred samples from the first group was freeze-dried for 4 h in a vacuum freeze dryer (LGJ-12A; China). The samples were then coated with gold for 2 min using a high-vacuum coating system (ACE600; Leica Microsystems, Germany). Subsequently, the surface morphology of the tobacco shreds was observed under a field emission scanning electron microscope (Verios 460; FEI Company, United States) at a scale of 5 µm.

### 2.4 Nontargeted analysis

Nontargeted analysis was performed using a Waters UPLC system coupled with a Sciex 5600 TOF mass spectrometer (MS). Chromatographic separation was achieved using a nine-gradient reverse-phase liquid chromatography (RPLC) method, following the detailed procedure outlined in our previous reports (Wang et al., 2023; Wang R.-Q. et al., 2025; Wang et al., 2025a). Specifically, separation was carried out on a Waters ACQUITY UPLC HSS T3 column (130 Å, 1.7 μm, 2.1 × 100 mm) with a binary mobile phase consisting of water and acetonitrile. Both phases contained 0.1% formic acid. The mobile phase flow rate was set at 0.3 mL/min, and the column temperature was maintained at 40 °C. A quality control (QC) sample was prepared by pooling equal volumes of all samples and was analyzed using the nine different LC gradients (Table 1). The fifth gradient (G5) was selected and employed for the analysis of each individual sample. The sample injection volume was set to 2 μL. Mass spectrometric analysis was conducted using a data-independent acquisition (DIA) method coupled with a data deconvolution algorithm. The TOF MS data were acquired across a mass range of 70–1,200 Da with a dwell time of 150 m. The DIA method utilized 12 variable SWATH windows, with each MS/MS scan lasting 50 m. Data were acquired in both positive and negative ion modes. The declustering potential (DP), collision energy (CE), and collision energy spread (CES) were set to 80 V, 35 V, and 15 V, respectively. Deconvolution of the collected DIA datasets was performed using a chromatographic retention behavior (CRB) algorithm for comprehensive data processing and analysis.

**TABLE 1:** Timing schedule for 9-gradient nontargeted screening in RPLC.

Gradient	Timing table				
G1	Duration (min)	0∼1.5	1.5∼5	5∼24	24∼27
	Water content (vol%)	99	10	2	2
G2	Duration (min)	0∼1.5	1.5∼10	10∼24	24∼27
	Water content (vol%)	99	10	2	2
G3	Duration (min)	0∼1.5	1.5∼15	15∼24	24∼27
	Water content (vol%)	99	10	2	2
G4	Duration (min)	0∼1.5	1.5∼20	20∼24	24∼27
	Water content (vol%)	99	10	2	2
G5	Duration (min)	0∼1.5	1.5∼23	23∼24	24∼27
	Water content (vol%)	99	10	2	2
G6	Duration (min)	0∼1.5	1.5∼23	23∼24	24∼27
	Water content (vol%)	99	30	2	2
G7	Duration (min)	0∼1.5	1.5∼23	23∼24	24∼27
	Water content (vol%)	99	50	2	2
G8	Duration (min)	0∼1.5	1.5∼23	23∼24	24∼27
	Water content (vol%)	99	70	2	2
G9	Duration (min)	0∼1.5	1.5∼23	23∼24	24∼27
	Water content (vol%)	99	90	2	2

## 3 Results and discussion

### 3.1 Cold plasma-induced changes in sensory profiles

**FIGURE 1:**
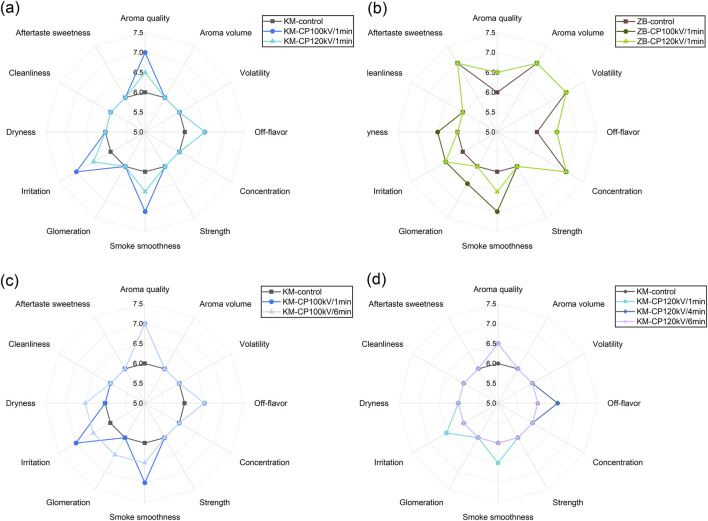
Radar plots of sensory attributes for Kunming (KM) and Zimbabwe (ZB) tobacco samples under different cold plasma (CP) treatments: **(a)** KM at 100 kV and 120 kV for 1 min; **(b)** ZB at 100 kV and 120 kV for 1 min; **(c)** KM at 100 kV for 1 min and 6 min; **(d)** KM at 120 kV for 1, 4, and 6 min.

CP treatment was found to influence the sensory attributes of both Kunming (KM) and Zimbabwe (ZB) tobacco leaves, with effects dependent on treatment voltage and duration. In KM tobacco, treatment at 100 kV for 1 min produced noticeable improvements in several sensory parameters (Figure 1a). Specifically, aroma quality increased from a baseline score of 6.0–7.0, while smoke smoothness and irritation also improved from 6.0 to 7.0. These enhancements suggest that low-voltage plasma can enhance the release or perception of desirable volatile compounds while simultaneously reducing harshness. However, when the voltage was increased to 120 kV, the benefits were slightly compromised. For example, aroma quality dropped from 7.0 to 6.5, and similar modest declines were observed in smoothness and irritation. These findings imply that while CP treatment is beneficial at lower intensities, excessive voltage may begin to degrade favorable compounds or introduce sensory fatigue.

In Zimbabwe tobacco, the response to CP treatment followed a similar but less pronounced pattern (Figure 1b). Aroma quality showed a small increase from 6.0 to 6.5 upon CP application at both 100 kV and 120 kV. Unlike KM tobacco, attributes such as aroma volume, volatility, and concentration were already high in the control samples and remained unchanged by CP treatment, consistently scoring 7.0. This suggests that Zimbabwe tobacco may possess a stronger intrinsic aromatic profile that is less responsive to CP stimulation. Improvements were still observed in terms of smoke smoothness and aftertaste sweetness, both of which increased following plasma exposure. Notably, off-flavor scores remained low or slightly improved, indicating that the treatment did not introduce any undesirable notes. The stability of strength, dryness, and cleanliness across all treatments further supports the conclusion that the effects of CP treatments are targeted and not broadly disruptive.

The time dependence of CP treatment was examined further on KM tobacco at both 100 kV and 120 kV (Figures 1c,d). At 100 kV, extending the treatment from 1 min to 6 min did not result in significant additional improvements (Figure 1c). Aroma quality remained at 7.0, and smoothness and irritation scores showed minor fluctuations without clear benefit. Interestingly, glomeration increased slightly at 6 min, potentially indicating structural or compositional changes with prolonged exposure (Figure 1c). In contrast, the 120 kV time series revealed a slight decline in sensory quality with longer treatment times (Figure 1d). While aroma quality reached 6.5 after 1 min and remained stable, smoke smoothness dropped from 6.5 to 6.0 after 6 min, and irritation and off-flavor showed similar patterns of slight deterioration. These results suggest that prolonged treatment at higher voltage can lead to over-processing, possibly by inducing oxidation or degradation of volatile and flavor-relevant molecules (Figure 1d).

Overall, CP treatment was effective in enhancing several key sensory attributes of tobacco, particularly in KM samples, which showed improvements in aroma and smoke character with low-voltage, short-duration treatments. Zimbabwe tobacco, due to its already high aromatic intensity, exhibited only modest additional improvements. Importantly, excessive treatment time or voltage appeared to reverse some of the benefits, emphasizing the need for careful optimization. These findings support the use of CP as a controllable post-harvest technology for improving tobacco quality, especially when tailored to specific leaf types and treatment parameters.

### 3.2 Cold plasma-induced changes in microstructures

**FIGURE 2:**
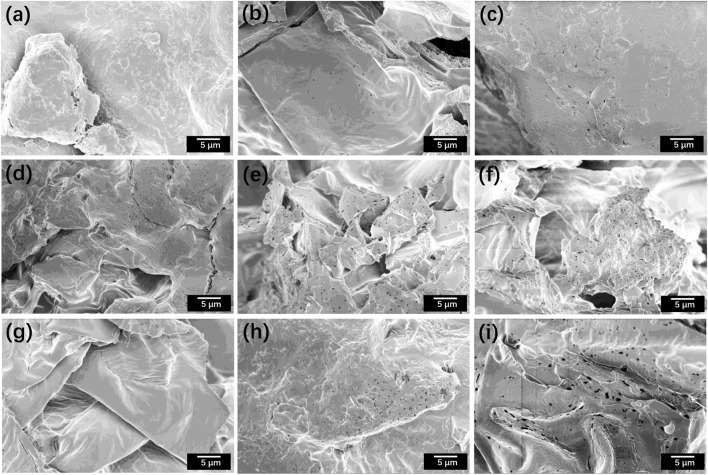
Scanning electron microscopy (SEM) images of tobaccos before and after CP treatments: **(a)** KM untreated; **(b)** KM at 100 kV for 1 min; **(c)** KM at 100 kV for 6 min; **(d)** KM at 120 kV for 1 min; **(e)** KM at 120 kV for 4 min; **(f)** KM at 120 kV for 6 min; **(g)** ZB untreated; **(h)** ZB at 100 kV for 1 min; **(i)** ZB at 120 kV for 1 min.

Scanning electron microscopy (SEM) images reveal the microstructural characteristics of tobacco samples from nine experimental groups (Figure 2). The untreated samples exhibited a compact and intact surface morphology (Figures 2a,g). In contrast, both Kunming and Zimbabwe tobaccos subjected to CP treatment showed progressively greater surface disruption with increasing voltage intensity, characterized by enlarged pores, increased pore density, lamellar separation, and surface wrinkling (Figure 2b–f, h–i). Longer treatment durations further intensified pore formation and cellular deformation. The effect of CP treatment on tobacco shreds is similar to the phenomena observed in previous studies (Bao et al., 2021; Chen X. et al., 2024). This effect accelerates moisture removal during drying, thereby enhance drying kinetics (Bao et al., 2021). Additionally, the disruptive effect of CP treatment on plant cell walls may promote the release of internal compounds (Chen X. et al., 2024).

These microstructural alterations can be attributed to the physical and chemical effects induced by CP treatment. At high-voltage CP conditions, strong electroporation effects disrupted cellular membrane integrity, generating reversible or irreversible pores in the cuticle and cell walls (Khudyakov et al., 2022). Simultaneously, reactive oxygen and nitrogen species (RONS) produced by CP, including hydroxyl radicals (•OH), N_2_ second positive systems, N_2_ first negative systems, and atomic N and N^+^, bombarded the sample surfaces at high velocities (Zhang et al., 2019). These species interacted with hydrogen bonds and other non-covalent interactions in cell wall components, resulting in pronounced etching effects and the formation of substantial cavities (Seelarat et al., 2024).

The resulting expansion of pore area and increase of pore density are expected to enhance thermal exchange during drying (Ashtiani et al., 2020). These structural changes not only contribute to improved drying efficiency but may also promote the release of flavor compounds from the tobacco matrix, potentially enhancing the quality of the final product.

### 3.3 Cold plasma-induced changes in molecular compositions

Nontargeted metabolomic profiling of tobacco leaves before and after cold plasma (CP) treatment detected 68,500 reproducible ion features, each with signal intensities at least threefold higher than those in blank controls. To identify treatment-responsive compounds, pairwise comparisons between treated and untreated samples were conducted using Student’s t-tests. Ion features were considered differentially abundant (DIFs) if they exhibited a fold change greater than 3 and a false discovery rate (FDR)–adjusted p-value less than 0.05 (i.e., q < 0.05). Across all CP conditions, KM consistently showed a greater number of decreases than increases (for example, 11,581 increased versus 17,069 decreased at 100 kV for 1 min, and 9,995 increased versus 20,203 decreased at 120 kV for 4 min; Table 2). In contrast, ZB showed a smaller net shift (i.e., 9,756 increased versus 10,077 decreased at 100 kV for 1 min; 8,502 increased versus 10,449 decreased at 120 kV for 1 min). This greater degree of chemical remodeling in KM aligns with sensory evaluation results, where mild, short CP treatment produced the most pronounced improvements in aroma and smoke character for KM. By comparison, ZB, which is already characterized by high aromatic intensity, exhibited only modest sensory gains.

**TABLE 2:** Number of differential Ion features in CP-treated tobacco leaves.

Sample code	Increased ion feature^a^	Decreased ion feature^a^
KM-100kV/1 min	11,581	17,069
KM-100kV/6 min	10,572	18,858
KM-120kV/1 min	10,132	19,539
KM-120kV/4 min	9,995	20,203
KM-120kV/6 min	11,765	19,740
ZB-100kV/1 min	9,756	10,077
ZB-120kV/1 min	8,502	10,449

^a^ The differential ion features are either 3 folds higher or lower and q < 0.05 in CP-treated tobacco leaves in comparison to untreated tobacco leaves.

**FIGURE 3:**
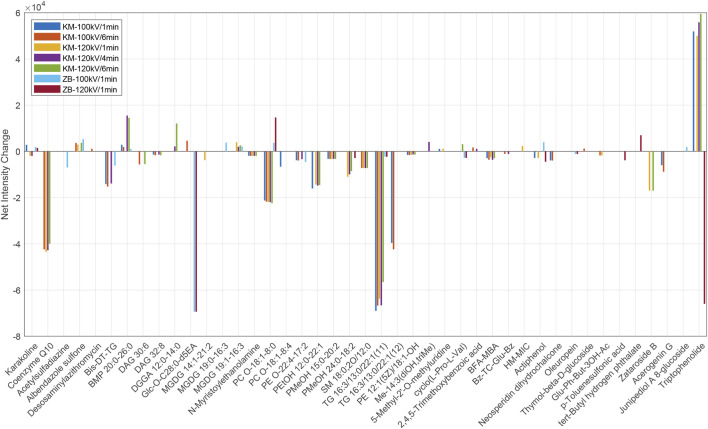
Net intensity changes of selected metabolites under seven treatment conditions. The bar chart shows the differences in compound intensities between treated and untreated samples across the CP treatment conditions. Compound names are shown as abbreviations for clarity. These include Bis-DT-TG (2,3-bis [[(3Z,6Z,9Z)-dodeca-3,6,9-trienoyl]oxy]propyl tridecanoate), TG 16:3/13:0/22:1 (11) and (12) (isomers of triacylglycerols), PE 12:1 (5Z)/18:1-OH ([2-[[(Z)-dodec-5-enoyl]amino]-3-hydroxyoctadecyl] 2-(trimethylazaniumyl)ethyl phosphate), Glu-Ph-But-3OH-Ac ([4-[3,4,5-trihydroxy-6-(hydroxymethyl)oxan-2-yl]oxyphenyl]methyl 3-acetyloxy-2-hydroxy-2-[(4-hydroxyphenyl)methyl]butanoate), HM-MIC (7-Hydroxy-3-(hydroxymethyl)-5-methylisochromen-1-one), BFA-MBA (2-(5-Acetyl-2,3-dihydrobenzofuran-2-yl)allyl 3-methylbutanoate), Bz-TC-Glu-Bz (2-(benzoyloxymethyl)-6-hydroxy-8-methyl-9,10-dioxatetracyclo [4.3.1.0^2^,^5^.0^3^,^8^]decan-3-yl]oxy]-3,4,5-trihydroxyoxan-2-yl]methyl benzoate), and Me-14:3 (diOH, triMe) (methyl (2E,4E,8E)-7,13-dihydroxy-4,8,12-trimethyltetradeca-2,4,8-trienoate).

A total of 43 putative metabolite annotations were assigned by matching differential features against established metabolite databases (Figure 3, and Supplementary Data Sheet 1). The confidence level of these annotations, according to the molecular species identification (MSI) levels raised by Schymanski et al., corresponds to level 2a (Schymanski et al., 2014). According to the putative annotations, it is found that the strongest sensory improvements corresponded to a distinct lipidomic profile that represents a chemical “sweet spot” for processing. Under mild CP treatment, particularly at 100 kV for 1 min, KM leaves showed marked yet incomplete depletion of bulky, highly unsaturated, and ether-linked membrane lipids. This group included plasmalogen-like phosphatidylcholines (e.g., PC O-18:1_8:0) and phosphatidylethanolamines (e.g., PE O-22:4_17:2), sphingolipids (e.g., SM 18:0; 2O/12:0), and the redox cofactor coenzyme Q10 (CoQ10). Concentrations of these compounds typically declined by over 70%. This selective reduction is consistent with the action of reactive oxygen and nitrogen species on unsaturated acyl chains and vinyl-ether linkages at the leaf surface (Ke et al., 2022). Mechanistically, it has been reported that lowering the abundance of hydrophobic lipids reduces the aerosolized tar fraction, diminishes oily or soapy mouthfeel, softens smoke harshness, and enhances the clarity of volatile aroma release (de Roos, 2005). These chemical adjustments are closely aligned with the sensory evaluation reports of brighter aroma, cleaner top-notes, and smoother smoke under mild CP conditions.

The data also clarify why exceeding the mild treatment regime leads to a decline in product quality. Prolonged or higher-intensity CP exposures resulted in pronounced increases in biochemical markers of chloroplast membrane degradation. These included bis(monoacyl)glycerophosphate (BMP 20:0_26:0, showing 5–6-fold increases), digalactosyldiacylglycerol-derived fragments (DGGA 12:0_14:0, absent under 120 kV/1 min treatment but abundant at 120 kV/4–6 min treatments), and monogalactosyldiacylglycerols (such as MGDG 19:1_16:3, which decreased at 120 kV/1 min but appeared *de novo* at 120 kV/4–6 min treatments). The accumulation of these polar lipid fragments, together with secondary oxidation products, is likely to impart grassy or oxidized flavor notes and reduce smoke smoothness (Grebenteuch et al., 2021). Furthermore, diketopiperazines associated with roasted or bitter sensory qualities, such as cyclo (L-Pro-L-Val), were detected only under the highest treatment conditions (KM, 120 kV for 6 min) (Stark and Hofmann, 2005). This chemical profile aligns closely with the sensory evaluations that excessive CP treatment produces a harsher and less pleasant smoking experience.

Changes in nitrogenous and small aromatic metabolites were also aligned with the observed sensory effects. N-acylethanolamide (i.e., (Z)-N-[3-hydroxy-1-[3,4,5-trihydroxy-6-(hydroxymethyl)oxan-2-yl]oxyheptacosan-2-yl]dodec-5-enamide, Glc-O-C28:0-d5EA) and N-myristoylethanolamine were completely depleted under all CP conditions in both tobacco varieties. Reducing this lipophilic nitrogen-containing species is likely to lower the formation of pyrolysis-derived amines, thereby diminishing acrid or harsh smoke notes (Senneca et al., 2007). In KM, several small phenolics and benzoates increased following CP treatments, such as 2,4,5-trimethoxybenzoic acid rose by 3.8–5.2-fold, and the phenolic lactone triptophenolide became detectable only after CP exposure. These compounds are essential for creating complex, appealing taste experiences (Schieber and Wüst, 2020; Souza et al., 2025). In contrast, ZB exhibited comparatively muted changes in these chemical classes, which is in line with its more modest sensory improvement.

The varietal context provides further insight into these differences. KM responds most favorably to treatment at 100 kV for 1 min, a condition that selectively reduces ether-linked and polyunsaturated phospholipids and sphingolipids to a degree sufficient to smooth smoke texture and enhance aromatic brightness, while avoiding excessive oxidation of chloroplast lipids. In contrast, when KM is exposed to 120 kV for 4–6 min, there is a marked accumulation of chloroplast-derived lipid fragments and diketopiperazines, which coincides with a decline in sensory quality (Serra Bonvehí and Ventura Coll, 2000; Chen Q.-Y. et al., 2024). For ZB, some ether phosphatidylcholines (PCs) even increase at 100 or 120 kV for 1 min (e.g., PC O-18:1_8:0 rising from non-detectable levels to 3,677–14,641), suggesting that this variety has both a lower requirement and a reduced capacity for lipid pruning. This likely explains why ZB, with its already favorable aromatic profile, exhibits only modest sensory improvements. Nonetheless, applying the same mild, short-duration treatment to ZB effectively avoids the over-processing chemical signatures that emerge at higher doses.

In conclusion, nontargeted analysis results provide clear guidance for process optimization. First, treatment should be maintained within the mild range, approximately 100 kV for 1 min, particularly for KM, to achieve an optimal balance between reducing heavy membrane lipids and preserving matrix components that facilitate desirable aroma release. Second, pronounced increases in bis(monoacylglycero)phosphate (BMP), digalactosyldiacylglycerol (DGGA), or monogalactosyldiacylglycerol (MGDG), together with the appearance of diketopiperazines, should be regarded as early warning indicators of over-oxidation and potential quality loss. Third, beneficial treatment can be tracked by monitoring consistent but mild reductions in ether phosphatidylcholines (PCs), phosphatidylethanolamines (PEs), and coenzyme Q10 (CoQ10), coupled with modest increases in small phenolics.

### 3.4 Verification with soy lecithin doped tobacco leaves

To validate the findings from the nontargeted metabolomic analysis, a verification experiment was conducted using soy phospholipids as a model lipid material. The previous analysis suggested that lipid composition plays a critical role in determining the flavor quality of tobacco, with mild CP treatment potentially modifying surface lipids to enhance sensory characteristics. To test this hypothesis, soy lecithin (SL) was applied to KM tobacco shreds to simulate an increased surface lipid load, and the effects of subsequent CP treatment were evaluated through both sensory assessment and nontargeted metabolomic profiling. This design enabled direct verification of whether CP treatment could mitigate the negative sensory effects induced by excess surface lipids, thus supporting the proposed lipid-mediated mechanism of flavor improvement.

**FIGURE 4:**
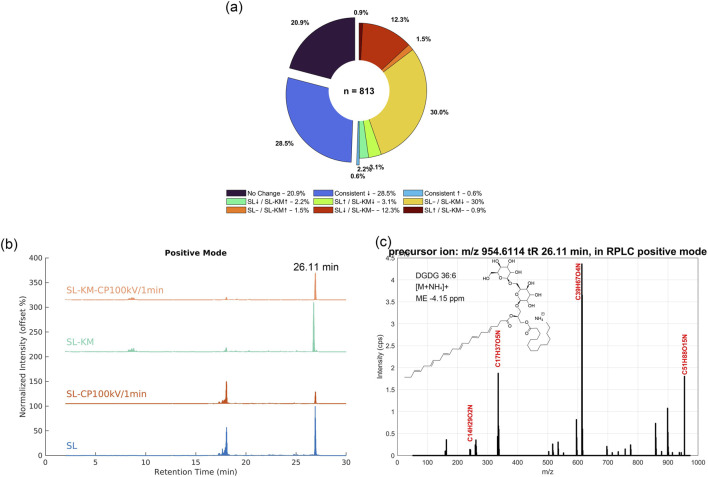
Metabolomic changes in pure soy lecithin (SL) and SL added to KM tobacco (SL-KM), after CP treatment at 100 kV for 1 min. **(a)** Nontargeted metabolomic profiling showing 813 trackable ion features, with intensity changes in SL and SL-KM following CP treatment. **(b)** Extracted-ion chromatogram (EIC) overlays of 569 positive ions with variable intensities before and after CP treatment. **(c)** MS/MS spectrum of the ion at m/z 954.6114, with labeled fragments and corresponding formulas, indicating a molecule with a structure similar to digalactosyldiacylglycerol (DGDG) 36:6.

The nontargeted analysis identified 813 trackable ion features (Figure 4a, Supplementary Data Sheet 2). Of these, 170 ions (20.9%) showed no significant change, while the remaining ions exhibited notable intensity changes in both SL and SL-KM following CP treatment. The majority of ion features (232 ions, 28.5%) showed a consistent decrease in intensity after CP treatment, likely reflecting the reduction or modification of lipid-related components, which aligns with the sensory perception of improved smoothness, reduced off-flavor and irritation. This decrease was particularly pronounced in the SL-KM group (244 ions, 30.0%) compared to the SL group, suggesting that CP treatment disrupts the interaction between surface lipids and the tobacco matrix, potentially removing or degrading specific molecules. On the other hand, 100 ions (12.3%) decreased in the SL group but remained unchanged in the SL-KM group, indicating that certain lipid components are protected from degradation when integrated with tobacco. Additionally, 43 ions (5.3%) exhibited contrary trends between SL and CP-treated SL, suggesting that CP treatment induces differential modifications depending on the presence of tobacco, highlighting the complex interactions between CP, lipids, and the tobacco matrix. Fewer ions showed increased intensities after CP treatment, including 7 ions (0.9%) that increased in SL but remained unaffected in SL-KM, 12 ions (1.5%) that increased in SL-KM but not in SL, and 5 ions (0.6%) that increased consistently in both SL and SL-KM. These results suggest that CP treatment primarily induces the decomposition or removal of surface lipids, rather than activating pathways related to the release or formation of desirable aromatic compounds. This process likely helps alleviate the unwanted sensory effects of SL-modified KM tobacco, such as irritation and off-flavor.

Among the 643 ions that exhibited intensity changes, a substantial majority, 569 ions (88.4%), were positive ions (Supplementary Data Sheet 2). To identify the components most affected by CP treatment, the Total Ion Chromatograms (TICs) of ions with variable intensities were analyzed (Figure 4b). A prominent component observed at 26.11 min showed high intensity, with substantial decreases following CP treatment, a pattern consistent across both SL and SL-KM samples. This component corresponds to a precursor ion with a mass-to-charge ratio (m/z) of 954.6114, which, based on its MSMS fragmentation pattern, was identified as a molecule with the molecular formula C_51_H_88_O_15_N ([M + NH_4_]^+^, ME -4.15 ppm, Figure 4c). The fragmentation data suggests that the molecule contains a long-saturated alkyl chain, C_14_H_29_O_2_N, a characteristic feature of lipids. Additionally, a diglycosidic moiety, identified as a disaccharide with a proton cleaved, is inferred from the neutral loss of C_12_H_21_O_11_ between the precursor ion and the characteristic fragment C_39_H_67_O_4_N. Further comparison with PUBCHEM chemical structures indicated that this ion is an ammonium adduct of a compound closely resembling digalactosyldiacylglycerol (DGDG 36:6) (Compound ID 134768992 in PUBCHEM). DGDG 36:6 is a galactolipid that is typically found in plant membranes (Rocha et al., 2018; Wang et al., 2020). The confidence level of this annotation corresponds to level 2b. This annotation level indicates a probable structure annotated through diagnostic fragments and parent compound information (Schymanski et al., 2014). The result suggests that the CP treatment may be targeting lipid components, particularly those with long-chain fatty acids, to alter their molecular structure. These findings provide further insight into the molecular mechanisms underlying the CP-induced changes in tobacco, specifically in relation to the modification of surface lipids that affect sensory characteristics. The consistent reduction in intensity of this component after CP treatment implies its potential role in mitigating the negative sensory effects of excess lipids, aligning with the observed improvements in aroma and smoothness in the sensory evaluation.

## 4 Conclusion

In conclusion, this study demonstrates that CP treatment effectively enhances the sensory quality of tobacco by modulating both its physical structure and chemical composition. The significant improvements in aroma quality, smoke smoothness, and reduced irritation following CP treatment support its potential as a valuable post-harvest processing technology for tobacco. SEM analysis confirmed that CP treatment induces microstructural changes, such as increased pore density and surface disruption, which may aid in moisture removal and enhance the release of internal aroma compounds. Nontargeted metabolomic profiling further revealed that CP selectively reduces certain lipid classes, particularly those with long-chain unsaturated fatty acids, contributing to a more desirable flavor profile. These findings highlight the potential of CP to improve tobacco quality while minimizing the degradation of flavor compounds. Future studies should explore the optimization of CP treatment parameters for different tobacco varieties and investigate its long-term effects on tobacco shelf life and quality. Ultimately, CP offers a novel approach for improving tobacco flavor and enhancing the overall quality of the final product.

## Data Availability

The datasets presented in this study can be found in online repositories. The names of the repository/repositories and accession number(s) can be found in the article/Supplementary Material.
